# Application of the Liver Maximum Function Capacity Test in Acute Liver Failure: A Helpful Tool for Decision-Making in Liver Transplantation?

**DOI:** 10.1155/2016/7074636

**Published:** 2016-05-04

**Authors:** Florian Wolfgang Rudolf Vondran, Carsten Schumacher, Kai Johanning, Björn Hartleben, Wolfgang Knitsch, Olaf Wiesner, Elmar Jaeckel, Michael Peter Manns, Juergen Klempnauer, Hueseyin Bektas, Frank Lehner

**Affiliations:** ^1^Department of General, Visceral and Transplant Surgery, Hannover Medical School, 30625 Hannover, Germany; ^2^Department of Anaesthesiology and Intensive Care Medicine, Hannover Medical School, 30625 Hannover, Germany; ^3^Institute of Pathology, Hannover Medical School, 30625 Hannover, Germany; ^4^Department of Respiratory Medicine, Hannover Medical School, 30625 Hannover, Germany; ^5^Department of Gastroenterology, Hepatology and Endocrinology, Hannover Medical School, 30625 Hannover, Germany

## Abstract

*Background*. Despite aggressive intensive medical management acute liver failure (ALF) may require high-urgency liver transplantation (LTx). Available prognostic scores do not apply for all patients; reliable tools to identify individuals in need of LTx are highly required. The liver maximum function capacity test (LiMAx) might represent an appropriate option. Referring to a case of ALF after* Amanita phalloides*-intoxication the potential of the LiMAx-test in this setting is discussed.* Presentation of Case*. LiMAx was performed in a 27-year-old patient prior to and after high-urgency LTx. In accordance with clinical appearance of hepatic encephalopathy, coagulopathy, and acute kidney failure, the LiMAx-test constituted a fulminant course of ALF with hardly any detectable metabolic activity. Following LTx with a marginal donor organ (95% hepatosteatosis), uptake of liver function was demonstrated by postoperative increase of the LiMAx-value. The patient was discharged from hospital on postoperative day 26.* Discussion*. ALF often is associated with a critical state of the patient that requires almost immediate decision-making regarding further therapy. Application of a noninvasive liver function test might help to determine the prognosis of ALF and support decision-making for or against LTx as well as acceptance of a critical donor organ in case of a critically ill patient.

## 1. Introduction

Acute liver failure (ALF) represents a potentially fatal complication of severe hepatic illness due to various reasons. Many patients suffering from ALF profit from aggressive intensive medical management; nonetheless, salvage of a substantial proportion of affected individuals still relies on orthotopic liver transplantation (LTx) [1, 2]. Since the clinical state of patients suffering from ALF can be most critical, short-term decision-making regarding further treatment might be vital [3]. In this setting, not only determination for or against LTx but also decision-making regarding acceptance of a critical donor organ in case of high-urgency- (HU-) LTx might be necessary. Since the available prognostic clinical scores do not apply for all patients [3], reliable tools to allow for identification of those individuals who need a liver transplant and those who can be managed with medical treatment to achieve full recovery after ALF thus are highly desired. The liver maximum function capacity test (LiMAx) is known to predict postoperative outcomes in liver surgery [4]. Regarding non-acetaminophen and non-*Amanita phalloides* toxin induced ALF first promising data has been reported using the LiMAx-test [5]. As this noninvasive measurement allows for rapid quantification of liver function selectively looking at the Cytochrome P450 1A2 metabolism, liver function also can be determined when conventional laboratory parameters are not meaningful as during coagulopathy requiring blood product substitution and toxin-induced release of liver enzymes.

**Figure 1:**
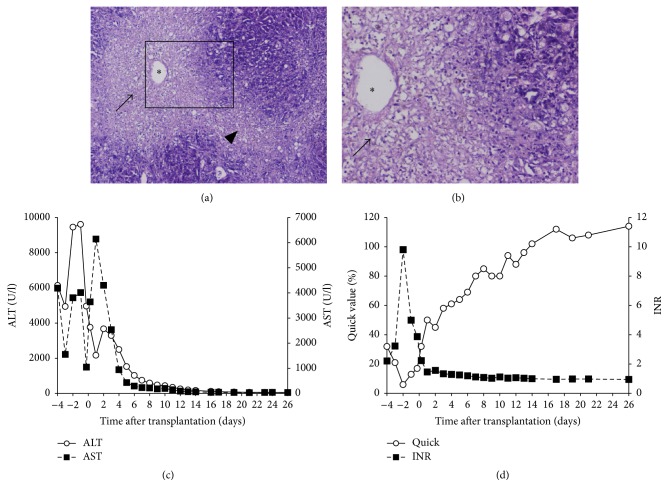
*Amanita phalloides* toxin can cause severe liver damage as observed in the explanted liver of our patient in terms of severe centrilobular necrosis (arrow) and bridging necrosis (arrowhead) of the liver epithelial cells. Asterisk marks central vein (HE staining; magnification: 40x and 100x, resp.) (a, b). This toxin-induced acute liver failure was associated with an appropriate increase of the liver transaminases (AST, ALT) (c) as well as coagulopathy depicted by decrease of the Quick value and increase of the INR (d).

*Amanita phalloides* is a wild mushroom that can cause severe liver damage in man (Figures 1(a) and 1(b)) clinically ranging from diarrhea to ALF. If not treated, mortality is as high as 80% [6]. Despite treatment (i.e., supportive measures, inactivation of the toxin) mortality still is 10–20% [7]. If ALF occurs, LTx so far seems to be the only therapeutic option, since the effectiveness of therapeutic concepts based on artificial liver devices is still suboptimal [8, 9]. Nevertheless, the criteria for LTx in ALF due to* Amanita phalloides*-intoxication are not consensual [10], and conventional laboratory parameters were not of any prognostic value [11].

Referring to a case of ALF after* Amanita phalloides*-intoxication the potential of the LiMAx-test in this setting will be discussed. Furthermore, the possible implication for the management of HU-LTx candidates in respect to the increasing number of critical donor organs will be pointed out.

## 2. Presentation of Case

We report on a healthy 27-year-old male patient who received HU-LTx due to ALF following* Amanita phalloides*-intoxication eating mushrooms collected by his girlfriend. Approximately 11 h after poisoning he suffered from diarrhea, vomiting, and abdominal pain. He was admitted to a local hospital where therapy with orally applied charcoal (25 g/d) and intravenous silibinin (20 mg/kg/body weight) was initiated immediately. Due to significant rise of transaminases (at norm on admission) and development of coagulopathy (Figures 1(c) and 1(d)) therapy was extended by acetylcysteine (5 g/d) as well as thiamine (300 mg/d) followed by transfer to our liver center the day after. Upon admission the patient presented with further aggravation of toxin-induced ALF (Figures 1(c) and 1(d)) but still no signs of hepatic encephalopathy. Therapy with charcoal, silibinin, acetylcysteine, and thiamine was continued. Urine screening 50 h after mushroom-ingestion still revealed positive* Amanita phalloides* toxin levels (1.6 *μ*g/L).

Despite continuation of the aforementioned therapy the patient's condition worsened continuously with requirement for intensive care treatment: transaminases further increased, coagulopathy worsened dramatically (Figures 1(c) and 1(d)), and relevant lactate levels (up to 14.9 mmol/L) were recorded; clinically he still suffered from diarrhea, whereas nausea, vomiting, and abdominal pain persisted; he developed hepatic encephalopathy grade II. Since the patient's medical history was free of further diseases, he thus was listed at Eurotransplant for HU-LTx with a MELD-score of 36 on day 4 after intoxication. He now received daily substitution with fresh frozen plasma (FFP) (4 units/d) and fibrinogen (2 g/d), but apart from mild hemorrhagic defecation no further bleeding events were observed. Due to development of acute kidney failure hemodialysis was begun.

On day 6 after toxin ingestion the patient received the first offer for LTx. The sighted deceased donor was a 59-year-old obese (body mass index of 28) female who suffered from a subarachnoidal bleeding due to a ruptured cerebral aneurysm. The donor liver was deemed steatotic by macroscopic appearance with confirmation by histology (60% micro- and 35% macrosteatosis). Since no other organ was available and the patient's condition was most critical, the organ eventually was accepted for transplantation following extensive interdisciplinary discussion.

**Figure 2:**
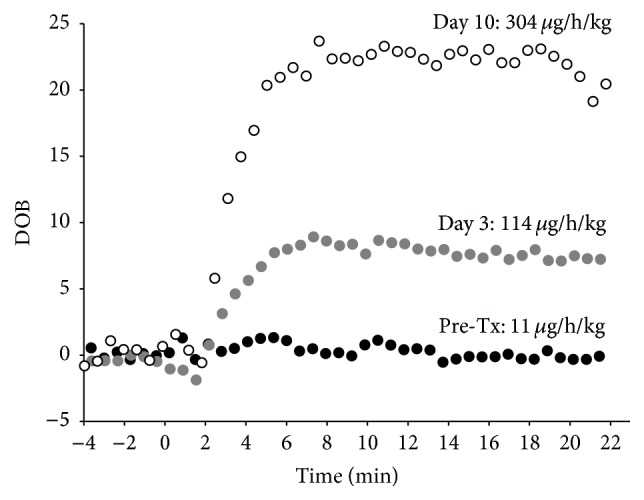
Measurement of the liver maximum capacity (LiMAx-test) revealed almost any metabolic activity at the time of transplantation (pre-Tx; black dots) but was seen to normalize in the course thereafter with almost normalized liver function at day 10 (white dots) after LTx (normal liver function = LiMAx value >315 *μ*g/h/kg; DOB: delta over baseline).

Waiting for the organ to arrive, the patient's liver maximum function capacity was determined applying the LiMAx-test (Humedics GmbH, Germany) by intravenously injecting a body weight-adapted dose of methacetin (2 mg/kg body weight) followed by detection of the exhaled ^13^C-labeled CO_2_ using the “Fast Liver Investigation Package” (FLIP) according to the manufacturer's instructions. By this means, onset of fulminant ALF was confirmed, as hardly any liver metabolism could be detected (Figure 2).

**Figure 3:**
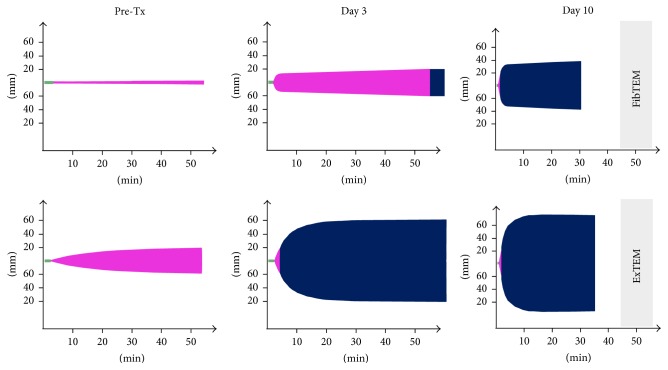
ALF also was observed performing pretransplant rotational thromboelastometry (ROTEM): ExTem clotting time (CT-Ex) was significantly prolonged (157 s) and maximum clot firmness in ExTEM (MCF-Ex) and FibTEM (MCF-Fib) reduced (20 and 3 mm, resp.). In line with the LiMAx-test, blood coagulation continuously recovered in the course of transplantation (CT-Ex of 51 s, MCF-Ex of 76 mm, and MCF-Fib of 37 mm at postoperative day 10, resp.).

In accordance with this finding severe derangement of blood coagulation was found performing rotational thromboelastometry (ROTEM) according to the manufacturer's instructions (Tem Innovations GmbH, Germany) and interpreted referring to published reference ranges [12] (Figure 3).

The patient was transplanted with the large (2422 g) steatotic organ of the aforementioned donor and a cold ischemic time of 7 h 37 min. LTx was performed with end-to-end vascular and biliary anastomoses. The warm ischemic time was 47 min. Due to diffuse bleeding the liver was packed and a planned second look was performed after 36 h. Triple immunosuppression (tacrolimus/mycophenolate mofetil/steroids) with anti-CD25 induction-therapy was provided. Histopathological work-up of the recipient's own liver demonstrated the severe liver damage caused by* Amanita phalloides* toxin (Figures 1(a) and 1(b)).

Despite known hepatosteatosis of 95% the graft showed an acceptable initial function. Only on postoperative day (POD) 1 mass substitution of blood products was performed (i.e., 18x FFPs, 12x packed red blood cells, 3x thrombocyte concentrates, 6 g Fibrinogen, and 3,000 units Antithrombin III). Thereafter no further substitution of blood products apart from Antithrombin III was necessary. The LiMAx-test and ROTEM analysis were repeated on POD 3 and POD 10, respectively, demonstrating continuous recovery of liver function in the course (Figures 2 and 3).

Following the second look operation on POD 2, the requirement for catecholamines (initially 0.181 *μ*g/kg/min norepinephrine) decreased continuously and the patient was free of catecholamines on POD 6. He quickly was weaned from the respirator (POD 6). Kidney function likewise rapidly improved following LTx with limited diuresis starting on POD 6 and cessation of hemodialysis on POD 9. Following bacteremia with detection of* Staphylococcus sciuri* but good response to an antibiotic treatment with Vancomycin (in the course switch to Ciprofloxacin), admission to the surgical ward eventually was performed on POD 11. The patient was discharged from hospital in good condition on POD 26.

## 3. Discussion

At present clinical scores are used for the management of patients suffering from ALF due to various reasons, with their respective limitations [3]. Reliable tools to exclusively monitor the liver function during treatment of ALF that simultaneously allow for valid prediction of prognosis of this condition hence are highly desired. In particular decision-making regarding the need for LTx but also defining the optimal time frame for organ replacement likewise would profit enormously by this means. The latter particularly applies for HU-LTx when critically ill patients receive offers from donors with marginal organs and an almost immediate decision is inevitable.

Primarily, the LiMAx-test has been developed for effective preoperative surgical risk evaluation to prevent major complications or even liver failure-related death. It has proven reliable in the assessment of liver function [13] and determination of postoperative outcomes in liver surgery [4], especially regarding avoidance of postoperative liver failure [14]. Impairment of liver recovery in case of nonalcoholic fatty liver disease could be demonstrated [15]. The LiMAx-test further was found to enable early diagnosis of sepsis-related hepatic dysfunction [16] as well as prognosis of early outcome after LTx [17]. Recently, a first small case series on the application of the LiMAx-test in patients suffering from ALF due to viral hepatitis, toxic liver injury (not* Amanita phalloides* induced), or cryptogenic liver failure likewise provided very promising results: whereas all biochemical parameters (i.e., bilirubin, creatinine, AST, ALT, and INR) were statistically indistinct, the liver maximum functional capacity test turned out to be of prognostic relevance with full recovery after ALF for a LiMAx value >38 *μ*g/kg/h [5].

Despite the great potential for the management of ALF and being a noninvasive breath test, the major hypothetical threat of the LiMAx-test in this setting also needs to be discussed: the intravenously applied substrate methacetin is metabolized by the liver to CO_2_ and acetaminophen. The latter is a well-known hepatotoxin that may lead to ALF itself when being overdosed [18]. The threshold for induction of ALF in healthy adults has been defined as a single dose of acetaminophen above 10 g or at least 200 mg/kg of bodyweight [19]. Whether the amount of acetaminophen that is set free during metabolism of methacetin may tip the scales regarding full recovery from or definitive development of ALF, or rather aggravate the patient's condition in ALF, remains questionable. It has to be noted though that the average amount of methacetin (applied at 2 mg/kg body weight) required to perform the LiMAx-test is equivalent to an acetaminophen dose of less than 50 mg [4] and thus severe impact on liver function by the LiMAx-test itself seems very unlikely.

Referring to the present case another issue related to transplantation can be discussed: following listing of a patient for HU-LTx it might be of great benefit to further define the time frame in which transplantation should be performed or, in other words, providing further evidence whether there is a need to accept a suboptimal organ to save the patient's life is highly desirable. In case of the young man listed for HU-LTx, we confirmed that ALF has taken a fulminant course applying the LiMAx-test (hardly any metabolic activity detectable). Within 48 h after listing of the patient a first offer for transplantation was obtained, which is in the usual range [20]. Nevertheless, due to the known shortage of donor organs [21] and the current strategy to at least partially counter the gap of supply by use of extended criteria donors (ECD) [22], only a marginal organ was available and the patient in most critical state. In line with data published by others [23], use of this ECD-organ in a good patient still resulted in an acceptable outcome. If the patient's liver maximum function capacity would have demonstrated a better residual liver function or longitudinal measurements would have indicated stable kinetics for the decrease of the liver function, one might have considered waiting for another and possibly better organ offer.

In conclusion, ALF—especially following* Amanita phalloides*-intoxication—often is associated with a critical state of the patient that requires almost immediate decision-making regarding further therapy. Application of a noninvasive liver function test might help to define the prognosis of ALF [5, 24], supporting not only determination for or against LTx but also decision-making regarding acceptance of a critical donor organ in case of a critically ill patient as demonstrated by the presented case.
